# Editorial: Psychological and neurocognitive mechanisms of presupposition processing in speech and language processing

**DOI:** 10.3389/fpsyg.2023.1289045

**Published:** 2023-10-30

**Authors:** Xiaolin Zhou, Xiaoming Jiang, Yingying Tan

**Affiliations:** ^1^Institute of Linguistics, Shanghai International Studies University, Shanghai, China; ^2^School of Psychology and Cognitive Science, East China Normal University, Shanghai, China; ^3^Key Laboratory for Language Sciences and Multilingual Artificial Intelligence, Shanghai International Studies University, Shanghai, China

**Keywords:** common ground, formal semantics and pragmatics, presupposition accommodation, presupposition triggers, neuropragmatics

When conveying a message, the speaker does not explicitly utter all the needed words and often uses presupposition to increase communicative efficiency (Schwarz, 2016). Linguistic presupposition refers to the use of certain lexical items or linguistic constructions (i.e., the so-called “presupposition trigger”) to encode certain unspoken meanings that typically exist in the shared knowledge, perceptions, beliefs, or attitudes between the speaker and the listener (Yang et al., 2022). These presupposition triggers (e.g. *even, also, still, again, if* , etc.) impose constraints on the use of certain mutual knowledge, which can be as strong as grammatical constraints in languages (Abusch, 2010; Yang and Jiang, 2023). Difficulty can arise when the constraints of using these linguistic expressions are not fulfilled (Jiang et al., 2009, 2013; Domaneschi et al., 2014; Jouravlev et al., 2016; Domaneschi and Di Paola, 2018; Zang et al., 2019). For example, in the sentence “Do you still play basketball?”, the speaker assumes that the listener has played basketball before. When this constraint (presupposition) is violated, for example if the listener did not play basketball before or for some reason did not know that they played basketball before, a difficulty arises in the listener when they hear this question.

To successfully understand the presupposed meaning, the listener has to derive underlying meanings by taking communicative contexts into account (Shetreet et al., 2019), resolving the ambiguous or underspecified interpretations (Schneider et al., 2021), and making pragmatic inferences beyond what is stated (Li et al., 2014). To achieve the capability of understanding such meanings, children must grasp certain maxims of language use through normal development of their pragmatic ability (Cheung et al., 2020).

While there has been progress in understanding the neurocognitive underpinnings of decoding a speaker's implied meaning over the past 20 years, exploration of the novel research direction of “neuropragmatics” has rarely focused on the mechanisms of presupposition processing. This Frontiers Research Topic includes seven original research articles on the issue, with several related questions raised and tackled. Among all the contributions, five studied the mechanisms of presupposition comprehension, and the other two focused on production strategies under certain pragmatic contexts.

## Linguistic and contextual factors modulate presupposition processing

Recent literature has marked the cognitive costs and benefits of recognizing presuppositions during language processing. Studies employing neurophysiological measures indicate the difficulty of integrating the presupposition trigger into the context, which leads to increased late ERP effects. However, it is unknown whether activating presupposition can benefit reading comprehension. The attenuating negative polarity items (NPI) are words that are used in contexts in which they weaken the assertion they appear. The attenuating NPI exerts a formal semantic constraint on vague and under-informative information (e.g., the phrase “all that” in English). Schwab and Liu showed that the use of attenuating NPI sounds more naturally when it is embedded in the premise conditional than in the indicative conditional. This finding demonstrates that the presupposition can alter the licensing of the use of certain linguistic terms by a formal semantic constraint.

A related question asks how the processing of presupposition interacts with other types of linguistic information. Li and Feng examined how informativeness interacts with the speaker identity in scalar implicature processing. Sentences of scalar implicature or *ad-hoc* implicature presuppose that the speaker describes the quantity of information in an informative way. Li and Feng showed that the underinformative description of scalar sentences is less accepted in L1 than in L2. These findings provide a novel case regarding how speaker identity information interacts with presupposition processing.

It is unclear how the mutual knowledge shared by the speaker and the listener is involved in the recognition of presupposition. Common knowledge between communicative partners is crucial for understanding presuppositions. Incentive structures of social contexts constrain whether the conversational partner is willing to share information with the speaker. The behavioral study on the response strategy of the conversational responder by Martín-Luengo et al. shows that the context encouraging the responder to only provide certain answers leads to a higher proportion of sharing responses compared with the context that maximizes providing the answers. This observation highlights how the listener's knowledge of the speaker modulates their pragmatic strategies in conversational communication.

## Communicative competency in conversations forms the prerequisite for presupposition processing

How does perspective shifting and other cognitive processes contribute to the derivation and verification of the presupposition processing? Using a developmental approach, Schidelko et al. targeted how perspective-taking could play a role in understanding presuppositions in a dialogue setting. The study demonstrates that at the age of four, children start to develop certain presupposition constraints on the question which requires them to understand another person's mental state.

Another related topic is whether the use of presupposition can be learned, given that the linguistic choice can be highly dependent on the communicative setting. The processing strategy can be crucial for syntactic priming, a tendency for the speaker to repeat the same linguistic structure exposed before. Alzahrani showed the benefit of syntactic priming when an Arabic speaker was instructed to guess (or predict) what a virtual communicator partner would say in a sentence. This syntactic priming effect was stronger than when the speaker was simply asked to repeat the sentence. Predictions during speaking can influence the choice of the linguistic structure (dative alteration vs. temporal phrase) which could be crucial in whether a speaker should choose to use a presupposition in a sentence or not.

## Tracking the time course of processing nonliteral meaning and its implication in presupposition processing

It is still unclear how the dynamic processes of presupposition processing (e.g., generating and verifying the presuppositions) take place, including the relation between the processing of presupposition and other types of nonliteral meaning. Two studies have shown how nonliteral expressions are processed in real-time, with the first examining the pun and the second the transferred epithet.

The retrieval of the less salient meaning in the homophone is crucial to nonliteral interpretation, in particular pun comprehension. The processing steps underlying the nonliteral interpretation can be reflected in the eye fixation patterns at different positions during reading and can be critically involved in the processing of presupposition. Zheng and Wang showed that processing the less-dominant interpretation of a homophone leads to an increased sense of humor as compared with processing its salient meaning. At the same time, the total fixation duration is longer on the homophone and shorter on the word immediately following the homophone in the former than in the latter condition. These findings demonstrate the dynamic impact of contextual information on recovering the non-literal meaning and shed light on the potential mechanisms underlying the processing of conflict between the presupposed meaning and the literal meaning.

The processing of presupposition involves the semantic computation of implicit meanings and the composition of sentential constituents. The Chinese transferred epithet is a phrase in which the modifier and the modified conflict with each other in meaning but such a conflict can be resolved by iconicity. By comparing phrases including a transferred epithet and those of literal meaning, Liao et al. showed an increased N400 ERP component that could reflect an increased effort in meaning composition between constituents with marked iconicity. This finding has implications for understanding how nonliteral interpretation is computed and integrated into sentence representation.

These unique contributions clearly advance our understanding of the presupposition processing and put forward novel questions that could encourage interdisciplinary works from psychology, linguistics, speech communication and cognitive neuroscience, among others.

## Author contributions

XZ: Writing—review & editing, Conceptualization. XJ: Writing—original draft, Conceptualization. YT: Writing—review & editing.
